# An Outlook on the Etiopathogenesis of Pulmonary Hypertension in HIV

**DOI:** 10.7759/cureus.27390

**Published:** 2022-07-28

**Authors:** Jaimee J Palakeel, Mazin Ali, Phani Chaduvula, Sanika Chhabra, Smriti Lamsal Lamichhane, Vaiishnavi Ramesh, Collins O Opara, Farhana Yaqoob Khan, Gargi Kabiraj, Humaira Kauser, Jihan A Mostafa

**Affiliations:** 1 Medicine, California Institute of Behavioral Neurosciences & Psychology, Fairfield, USA; 2 Neurology, California Institute of Behavioral Neurosciences & Psychology, Fairfield, USA; 3 Telegeriatrics, Michigan State University, Grand Rapids, USA; 4 Family Medicine, California Institute of Behavioral Neurosciences & Psychology, Fairfield, USA; 5 Radiation Medicine, California Institute of Behavioral Neurosciences & Psychology, Fairfield, USA; 6 Pathology, California Institute of Behavioral Neurosciences & Psychology, Fairfield, USA; 7 Internal Medicine, California Institute of Behavioral Neurosciences & Psychology, Fairfield, USA

**Keywords:** inflammatory mediators, hiv-hcv co-infection, hhv-8, hiv-nef, hiv-tat, hiv gp120, bmp-2, nadph, pulmonary arterial hypertension, ros

## Abstract

Although overall survival rates of patients infected with human immunodeficiency virus (HIV) have been significantly improved by antiretroviral therapy (ART), chronic comorbidities associated with HIV result in a worsening quality of life. Pulmonary arterial hypertension (PAH) is the most prevalent comorbidity associated with HIV infection. Despite low viremia and a non-replicative state maintained by ART, few people develop PAH. Previous data from animal models and human pulmonary microvascular endothelial cells (HPMVECs) suggests a constellation of events occurring during the propagation of HIV-associated PAH (HIV-PAH). However, these studies have not successfully isolated HIV virions, HIV-DNA, protein 24 antigen (p24), or HIV-RNA from the pulmonary endothelial cells (ECs). It provides an insight into an ongoing inflammatory process that could be attributed to viral proteins. Several studies have demonstrated the role of viral proteins on vascular remodeling. A composite of chronic inflammatory changes mediated by cytokines and growth factors along with several inciting risk factors such as Hepatitis C virus (HCV) co-infection, genetic factors, male predominance, illegal drug usage, and duration of HIV infection have led to molecular changes that result in an initial phase of apoptosis followed by the formation of apoptotic resistant hyperproliferative ECs with altered phenotype. This study aims to identify the risk factors and mechanisms behind HIV-PAH pathobiology at the host-pathogen interface at the intracellular level.

## Introduction and background

Although antiretroviral therapy (ART) plays a significant role in reducing the viral load, improving CD4 levels, mitigating mortality rates, and prolonging life expectancy, people on treatment still face a worsening quality of life due to the chronic complications of the human Immunodeficiency virus (HIV) [1-3]. Pulmonary arterial hypertension (PAH) is the most prevalent life-threatening disease associated with HIV, leading to death. PAH is defined as an elevated mean arterial pulmonary pressure of more than 25 mmHg with a pulmonary capillary wedge pressure of less than 15 mmHg and a pulmonary vascular resistance of more than 3 Wood units [2,4]. HIV-associated PAH (HIV-PAH) is categorized among group I classification of PAH with an overall prevalence about 0.5% higher than idiopathic PAH in the general population [4,5]. Patients with PAH may be asymptomatic or present with exertional dyspnea, extreme fatiguability, pedal edema, chest pain, syncope, or non-productive cough [4]. Asymptomatic patients are diagnosed at the most advanced stages, rapidly progressing to death. The three-year survival rate of patients with HIV-PAH varies depending on the New York Heart Association (NYHA) classification. On average, the survival rate is approximately 72%. Those in classes I and II, however, have a better survival rate (90%) than classes III and IV (<30%) [5].

Several studies on animal models and in vitro human pulmonary microvascular endothelial cells (HPMVECs) suggest possible mechanisms of the pathogenesis of HIV-PAH. However, there is no definitive evidence linking HIV and PAH [5]. We know that HIV type 1 (HIV-1) effectively replicates in the lungs [6]. However, HIV virions, HIV-DNA, protein 24 antigen (p24), or HIV-RNA have not been isolated from the endothelium of pulmonary vasculature in patients with PAH [7-9]. Moreover, despite low viral titers and a lack of competent replicative virus, patients on ART show an ongoing inflammatory process in the pulmonary vasculature [3]. These findings may implicate that the circulating HIV proteins indirectly mediate the release of inflammatory cytokines and growth factors resulting in endothelial dysfunction [7,8,10]. Also, ART may be ineffective in treating latent reservoirs [11]. Since only a small percentage of patients with HIV develop PAH, perhaps individual genetic predisposition may be important. The purpose of this review is to examine all potential risk factors and mechanisms at the host/pathogen interface that contribute to PAH at the intracellular level.

## Review

Host immune system and HIV persistence

**Figure 1:**
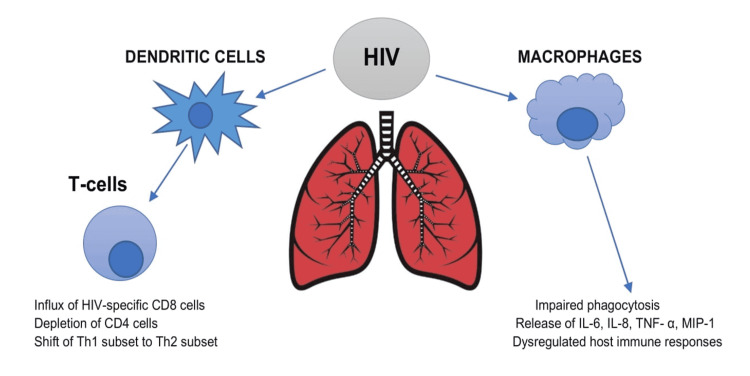
illustrates the effect of HIV on host immune cells HIV targets both the dendritic cells and alveolar macrophages, causing T-cell activation. This ultimately results in an increased influx of CD8 cells with depletion of CD4 cells, leading to T-cell exhaustion. There is also a shift from the Th1 subset to the Th2 subset, altering cytokine release. The infected alveolar macrophages result in impaired phagocytosis, the release of inflammatory cytokines, and dysregulated immune system [11]. Th: T helper cell; IL: interleukin; MIP-1: macrophage inflammatory protein-1; TNF-α: tumor necrosis factor-alpha, HIV: human immunodeficiency virus Image credit: Jaimee Jacob Palakeel

HIV affects the host immune system by invading the lungs. Firstly, it invades the bronchial epithelium, which functions as a barrier to the external environment. HIV directly infects dendritic cells, known as the antigen-presenting cells, resulting in T-cell activation. The virus attacks the lung's primary immune cell - alveolar macrophages - leading to impaired phagocytosis, dysregulated immune response, and viral persistence. The adaptive immune system is also primarily impaired due to decreasing CD4 levels and HIV-specific CD8 sequestration, causing T-cell exhaustion [11,12]. Secondly, HIV shifts the T helper cells (Th) from the Th1 subset to the Th2 subset. Th1 cells release interleukin (IL) such as IL-2 and interferon-gamma (IFN-γ), whereas Th2 cells produce IL-4, IL-5, IL-6, IL-10, and IL-13, which in turn inhibits the Th1 cytokines. During the HIV infection, a transition occurs, altering the cytokine release. As a result, the T-cell responses to the viral antigens and the cytotoxic T-cell lymphocytic activity decreases [13]. The IL-4 and IL-13-induced Th2 response leads to pulmonary artery muscularization and vascular remodeling [14]. Thirdly, HIV interferes with the migration of circulating host immune cells to the lungs [15]. HIV persistence results in chronic immune activation and a systemic inflammatory response mediated by IL-6, IL-8, macrophage inflammatory protein-1 (MIP-1), and tumor necrosis factor- α (TNF-α). Over time, chronic lung damage and injury are caused by the release of matrix metalloproteinases (MMPs) and intracellular adhesion molecules-1 (ICAM-1) from the lungs. Figure 1 explains the effect of HIV on the host immune system within the lungs. 

Table 1 summarizes the overall mechanisms involved in HIV-PAH.

**Table 1:** shows an overall summary of mechanisms involved in HIV-PAH Tat: transactivator of transcription; Nef: negative factor: gp120: glycoprotein 120; EC: endothelial cell; SMC: smooth muscle cell; eNOS: endothelial nitric oxide synthase; VEGF: vascular endothelial growth factor; PDGF: platelet-derived growth factor; EGF: epidermal growth factor; FGF: fibroblast growth factor; HIF-1α: hypoxia inducing factor-1α; NF-κB: nuclear factor kappa B; TNF-α: tumor necrosis factor- α; AP-1: activator protein-1; NOX: NADPH oxidase enzyme; MMP: matrix metalloproteinases; HCV: hepatitis C virus; HHV-8: human herpesvirus-8; BMPR-2: bone morphogenetic protein receptor-2; VCAM: vascular cell adhesion molecule; ICAM: intercellular adhesion molecule; CX3CL1: chemokine C-X3-C motif ligand 1; CCL2: C-C motif chemokine ligand 2; NADPH: nicotinamide adenine dinucleotide phosphate; HIV: human immunodeficiency virus; PAH: pulmonary arterial hypertension; ROS: reactive oxygen species Table credit: Jaimee Jacob Palakeel

SI No	Factors influencing the pathogenesis of HIV-PAH	Mechanisms
1.	HIV proteins - Tat/Nef/gp120	Apoptotic resistant ECs/ Pro-survival state/Angiogenesis/ reactive oxygen species/ decreased eNOS expression and NO resulting in vasoconstriction and vascular remodeling.
2.	Inflammatory cytokines and Growth factors	1. Release of IL-6, IL-1β, TNF-α, and macrophage inflammatory protein- 1α 2. Growth factors such as VEGF/PDGF/EGF/FGF and chemokines CX3CL1 and CCL2, increase VCAM/ICAM expression, leading to monocyte recruitment/inflammation/angiogenesis/repercussion of vascular injury.
3.	NOX enzymes and ROS	1. Formation of reactive oxygen species 2. Activation of transcription factors such as NF-kB, AF-1, HIF-1α, and TNF-α on the vasculature, promotes dysregulated apoptosis and proliferation, leading to migration of SMC mediated by metalloproteinases MMP-2 and MMP-9.
4.	Viruses	HCV, HHV-8
5	Genetic factors	HLA-DR6 and HLA-DR52, BMPR-2 mutation
6.	Illicit drugs	Cocaine/Morphine down-regulates BMPR-2 expression, causes phosphorylation of VEGF, promoting angiogenesis, smooth muscle proliferation, and vascular remodeling.

Indirect role of HIV proteins on pulmonary vasculature

Although the pathogenesis of HIV-PAH is unclear, data from animal models and in vitro human pulmonary microvascular endothelial cells (HPMVEC) implicate the role of HIV viral proteins - namely, transactivator of transcription (Tat), glycoprotein 120 (gp120), and negative factor (Nef) [16].

*HIV Tat* HIV-transactivator of transcription (Tat) is a viral protein that is integral to HIV-1 transcription. Tat is secreted by the infected cells and taken up by healthy bystander cells through a cell-penetrating peptide within its core domain [2,3]. It is then trafficked to the nucleus, where the transcription elongation factor of the host is recruited to the transactivation response element (TAR) in the RNA, facilitating HIV-1 transcription [16]. Besides its role intracellularly, it works in the extracellular milieu, binding to several receptors. Most importantly, it attaches to vascular endothelial growth factor-A (VEGF-A) tyrosine kinase receptor, regulated by fetal liver kinase-1/kinase insert domain receptor (Flk-1/KDR), triggering the integrin and fibroblast growth factor (FGF) pathways, thereby promoting angiogenesis. Its interaction with alpha-v beta-3 integrins (αvβ3) upregulates focal adhesion kinase activity and nuclear factor kappa B (NF-κB), leading to endothelial cell (EC) proliferation [16,17].

Tat protein is associated with the DNA damage response network. It interacts with a Tat interacting protein 60KDa (Tip60), a histone acetyltransferase involved in DNA repair. Tat acts by inhibiting the activity of Tip60, which in turn affects the phosphorylation of Ataxia telangiectasia mutated (ATM) kinases preventing caspase-mediated apoptosis and inducing a pro-survival cellular state. The Tat protein is also known to downregulate the DNA-protein kinase catalytic subunit (DNA-PK cs), which favors the repair of damaged DNA. As a result, cells accumulate excessive damaged DNA, producing reactive oxygen species (ROS) [2]. These ROS stimulate the release of hypoxia-inducible factor-1α (HIF-1 α) within the pulmonary EC, resulting in increased expression of smooth muscle mitogen and platelet-derived growth factor (PDGF), leading to smooth muscle cell (SMC) proliferation and progression to PAH. In contrast, there is decreased expression of endogenous antioxidant, superoxide dismutase, a mitochondrial superoxide scavenger with reduced glutathione levels [16]. A Tat transgenic mouse model demonstrated the influence of Tat on enhanced oxidative stress burden in the lungs and the downregulation of oxidative stress response transcription nuclear factor erythroid-2-related factor 2 (Nrf2) in the human primary pulmonary arterial ECs [2]. Studies conducted on ECs treated with Tat protein and cocaine showed a significant ROS level within the HPMVECs when treated together than ECs receiving either cocaine or Tat protein [17].

HIV-gp120

HIV-gp120 is an envelope protein on the surface of HIV that enables viral attachment and fusion to the host cell membrane [16]. HIV binds to CD-4 receptors on the surface of susceptible host cells such as Th cells and macrophages via gp120. Once binding occurs, gp120 undergoes a conformational change, exposing co-receptors on gp120 to bind to chemokine C-X-C type 4 receptor (CXCR4) / C-C chemokine receptor 5 (CCR5) co-receptors on the host immune cells. The HIV virions which bind with CCR5 are known as R5 viruses, while those attached to CXCR4 are X4 viruses [16,18]. ECs are seemingly resistant to HIV infection [6,19] since HIV virions, HIV-DNA, p24 antigen, or HIV-RNA have not been isolated from the pulmonary ECs [7-9,18]. However, a study performed by Sharilyn Almodovar *et al. *confirmed the presence of co-receptors in the HPMVECs. Extensive cellular damage mediated via the release of inflammatory cytokines, apoptosis, and tight junction injury, has been linked to gp120 binding [20]. The study emphasized the interaction between the X4 virus and CXCR4 in inducing pulmonary vascular inflammation. The expression of arachidonate 5-lipoxygenase (ALOX5) was increased, which led to a leukotriene-induced alteration in vascular permeability and vasoconstriction - both pathognomic changes associated with PAH. The study also showed that the R5 virus promoted apoptosis in ECs, while the X4 virus resulted in apoptotic resistance with proliferative EC phenotypes [18].

HIV-Nef

The HIV-Nef is an accessory protein expressed abundantly in the early phase of infection [21]. It plays a significant role in HIV persistence and immune system evasion. Nef is located in the cytoplasm and inner cell membrane but is secreted in the extracellular vesicle [2,16]. Intracellularly, Nef blocks the trafficking of the major histocompatibility complex-1 (MHC-1), thereby permitting the escape of infected cells from immune surveillance. Extracellularly, it is taken up by T-lymphocytes leading to suppression and depletion of CD-4 cells. It also infects macrophages, causing the release of MIP-1, IL-1β, IL-6, and TNF-α [16,22]. The conviction involved in the pathogenesis of HIV-PAH is characterized by an initial phase of pulmonary EC apoptosis followed by uncontrolled EC proliferation [16]. Studies have also shown that EC signal HIV-infected CD-4 cells mediated by Nef proteins even in the absence of active viral infection [23], as there is no evidence to support the direct EC-HIV infection [7].

**Figure 2:**
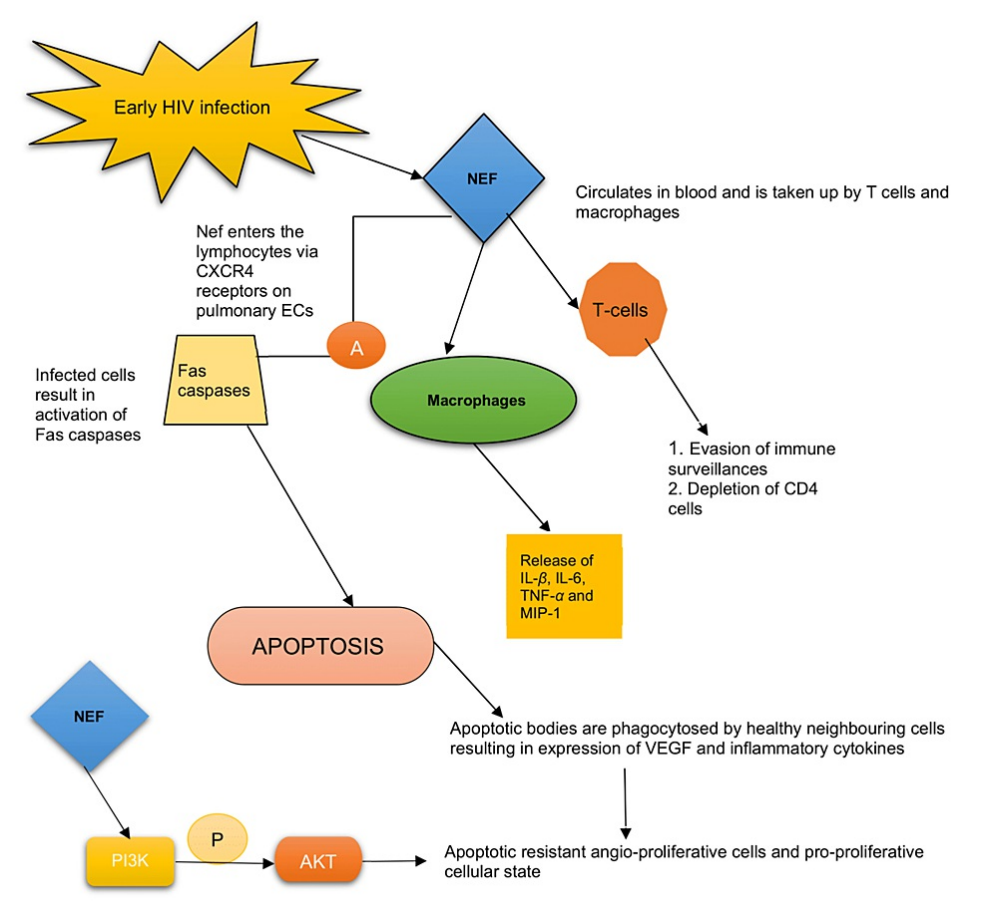
The role of Nef protein in the pathogenesis of HIV-PAH VEGF: vascular endothelial growth factor; MIP-1: macrophage inflammatory protein-1; TNF-α: tumor necrosis factor-alpha; PI3K: phosphoinositide 3-kinases; AKT: serine/threonine kinase (protein kinase B or AKT) pathway; P: phosphorylation; A: activation; HIV: human immunodeficiency virus; PAH: pulmonary arterial hypertension; Nef: negative factor Image credit: Jaimee Jacob Palakeel

Nef enters the lymphocyte via the CXCR4, expressed on pulmonary ECs causing apoptosis [16] - a mechanism mediated by activated Fas caspases (Fas is a member of the TNF-receptor family) [24]. The apoptotic bodies are then phagocytosed by neighboring uninfected cells that trigger the release of inflammatory cytokines and growth factors such as vascular endothelial growth factor (VEGF), leading to the formation of apoptotic resistant cells [21]. Ultimately, a dysregulated, uncontrolled EC proliferation and transition of the EC phenotype to an apoptotic-resistant angio-proliferative cellular state occurs [2,16,21,22]. There is also phosphorylation of the serine/threonine kinase AKT (or protein kinase B) pathway mediated by Nef-driven activation of phosphoinositide-3-kinases (PI3K), which leads to anti-apoptosis and creates a pro-proliferative cellular state [2]. In HPMVECs, Nef also diminishes endothelial nitric oxide synthase (eNOS) expression and promotes oxidative stress [25]. Figure 2 illustrates the role of Nef protein in mediating PAH.

The macaques model with simian immunodeficiency virus containing a chimeric viral construct with HIV-1 Nef gene (SHIV Nef virion) showed the presence of complex plexiform-like lesions noted in the pulmonary vasculature, characterized by narrowing of the lumen, EC proliferation, medial hypertrophy, thrombosis, and luminal recanalization [10,21,26]. These findings were absent in animals infected with naive simian immunodeficiency virus (SIV-Nef) [16,21,27]. It also displayed aberrant fragmented Golgi bodies in the cells affected by Nef associated with the dispersal of Golgi tethers giantin and p115 in plexiform lesions. This infers that Nef could jeopardize the integrity and function of the Golgi apparatus [16] but why HIV-Nef and not SIV-Nef affects the Golgi and how it contributes to PAH is not clearly understood [28]. Nef protein was identified on the pulmonary endothelium suggesting its vital role in the vascular remodeling in HIV-PAH [2,21,27]. To fully understand the pathogenesis of HIV-PAH, however, is to appreciate the source of Nef entirely [21].

Nicotinamide adenine dinucleotide phosphate (NADPH) oxidase-mediated generation of ROS: a culprit ensuing PAH

HIV infection has a deleterious effect on the alveolar macrophages by impairing their phagocytic role. It is assumed to be due to enhanced pulmonary oxidative stress [29]. It has been documented that oxidative stress disrupts the vascular tone and permeability through dysregulation of endothelial functions leading to vascular remodeling and the development of PAH [30]. Several pre-clinical and clinical studies have highlighted several mechanisms by which oxidative stress can occur within the pulmonary system [29]. In the lungs, the reduced form of NADPH oxidase, NOX, is considered the main element in generating ROS. NOX enzyme expressed in the vasculature consists of four isoforms, of which NOX1/NOX2/NOX4 plays a pivotal role in the pathogenesis of PAH. NOX enzyme results in dysfunction of the ECs, promoting inflammation and apoptosis [30]. The HPMVECs express NOX1 within the airway, alveolar epithelium, and ECs, NOX2 is expressed on the plasma membrane of the alveolar macrophages and ECs, and NOX4 in the mitochondria, endoplasmic reticulum of the smooth muscles, fibroblasts, and ECs. NOX1/NOX2 enzymes produce superoxide free radicals, while NOX4 enzymes form hydrogen peroxide/superoxide radicals [30,31]. Table 2 shows the sources of NOX enzymes within the respiratory system.

**Table 2:** Source of NOX enzymes within the lungs NOX: nicotinamide adenine dinucleotide phosphate (NADPH) oxidase enzyme Table credit: Jaimee Jacob Palakeel

SL NO	Type of NOX enzymes in lungs	Expression of NOX within lungs
1	NOX 1	Airway and alveolar epithelium, endothelial cells
2	NOX 2	Alveolar macrophages, dendritic cells, Smooth muscle cells, fibroblast, endothelial cells
3	NOX 4	Smooth muscle cells, fibroblasts, endothelial cells

**Figure 3:**
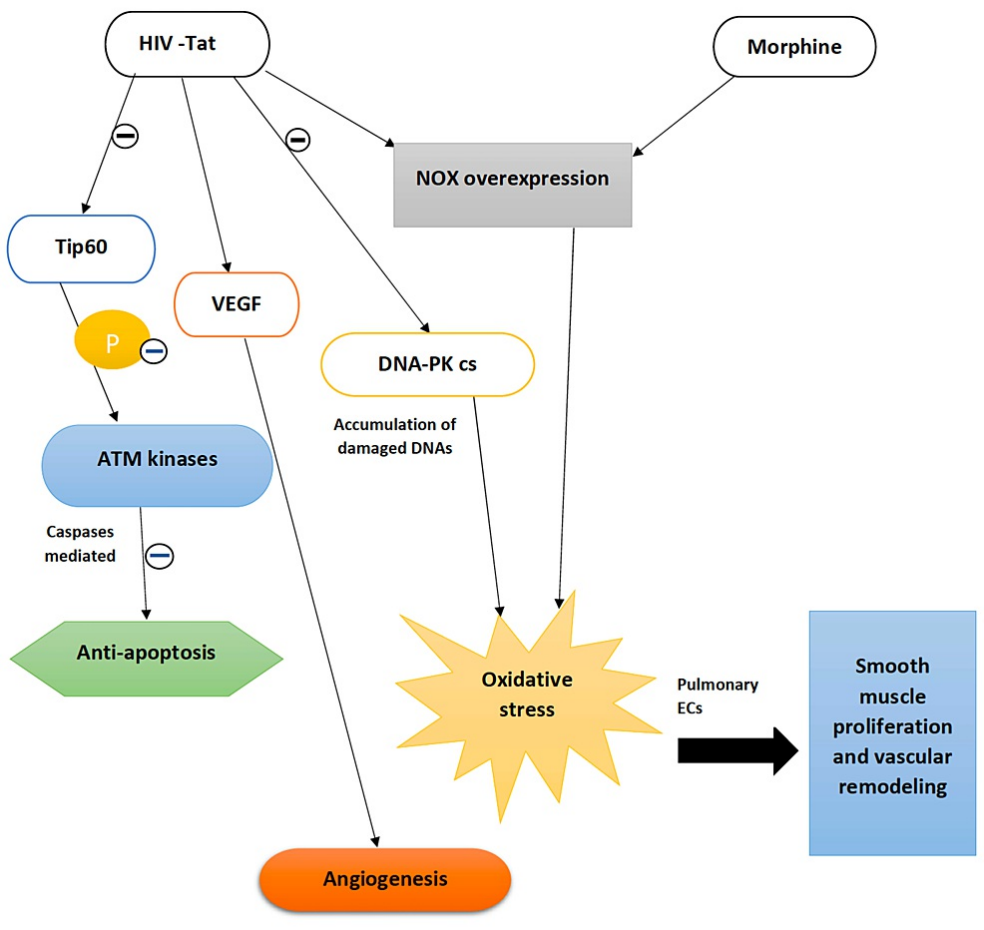
The effect of HIV-Tat protein and morphine on the pulmonary vasculature HIV-Tat interacts with Tip60 protein, a histone acetyltransferase, preventing the phosphorylation of ATM kinases and caspase activation, and leading to anti-apoptosis [2]. It results in the upregulation of the VEGF receptor, ensuing angiogenesis [16]. Both Tat and morphine cause overexpression of NOX enzymes that induce oxidative stress [30] on the pulmonary ECs, resulting in smooth muscle migration, proliferation, and vascular remodeling [16,20]. Likewise, Tat protein inhibits the activity of DNA-PK cs, the DNA repair protein, leading to the accumulation of damaged DNA and oxidative stress [2] Tat: transactivator of transcription; Tip60: Tat interacting protein 60KDa; ATM: ataxia telangiectasia mutated; VEGF: vascular endothelial growth factor; NOX: NADPH oxidase enzyme; ECs: endothelial cells; DNA-PKcs: DNA protein kinase catalytic subunit; P: phosphorylation; HIV: human immunodeficiency virus Image credit: Jaimee Jacob Palakeel

Analyses of in vitro studies have shown that HPMVECs, when subjected to chronic exposure to HIV-Tat or drugs like morphine, cause a transition in autophagy-dependence from the grade of initial apoptosis to an apoptosis-resistant hyperproliferative state [30,32]. The primary source of oxidative stress and free radical generation remains unclear. Studies on pulmonary ECs showed an initial apoptosis phase, which was accelerated when cells were exposed to HIV-Tat and morphine, leading to overexpression of the NOX2 enzyme. ROS formation was elevated in the early stage, indicating EC death. But on chronic exposure to insults such as morphine and HIV-Tat on HPMVECs, which resulted in NOX4 expression, the ROS showed a declining trend that favored a cell pro-survival state. ROS signals both apoptosis and cell proliferation in the endothelium depending on its concentration and cell type. As various studies have stated, the high concentration of free radicals on ECs leads to an apoptotic state due to grievous damage to the DNA, protein, and lipids signaling pro-apoptotic pathways. Low, non-toxic levels induce cell signaling events, more precisely, a cell proliferative phase [30]. Figure 3 explains the combined effect of HIV-Tat and morphine in vascular remodeling.

Effect of ROS on pulmonary vasculature

Pulmonary vascular remodeling, the pathobiological change contributing to PAH, initiates with changes in the ECs [33]. Evidence has shown that HIV-1 infection does not directly increase ROS production within the pulmonary endothelium. But its viral proteins, namely Tat and gp120, significantly contribute to this role. These proteins are released from HIV-infected cells like macrophages and T-cells and affect the uninfected neighboring cells via apoptosis [17]. The pulmonary vascular remodeling process is collectively brought about by various stimuli such as physical or chemical factors that significantly influence the generation of ROS. These could be mechanical stimuli, inflammatory cytokines, hypoxia, and growth factors. The most important of all will be the NADPH oxidase system [34].

It disrupts the tight junction proteins between the ECs, thereby altering the paracellular permeability, decreasing the endothelium's transepithelial resistance potential, and ultimately leading to the disintegration of the pulmonary arteries [17]. Oxidative stress creates an imbalance between oxidative enzymes and endogenous antioxidants in our bodies. The endothelial nitric oxide (NO) released through eNOS phosphorylation regulates the vascular tone and permeability through its vasodilatory effects and maintains an anti-proliferative anti-apoptotic state [33]. Oxidative stress decreases the activity of eNOS, compromising NO bioavailability and NO-dependent pulmonary vascular relaxation, thereby increasing pulmonary vascular tone [35]. In an eNOS knockout mouse that developed PAH, subsequently delivering the eNOS gene back to the lungs reversed PAH [36]. ROS also regulates the activity of transcription factors such as NF-κB, activator protein-1 (AP-1), HIF-1α, and TNF-α on the vasculature. This leads to dysregulated apoptosis and proliferation, causing migration of vascular SMCs mediated by matrix metalloproteinases (MMP-2 and MMP-9) [20]. Besides that, several in vitro studies have shown that the intracellular glutathione, an endogenous antioxidant, is lowered during oxidative stress [20,37], facilitating the activation of the NF-κB pathway and favoring HIV replication [20,38,39]. With active HIV replication, the major factors of lipid peroxidation, namely, malondialdehyde (MDA) levels and nitrotyrosine, are enhanced. This impact of ROS-induced viral replication leads to extensive vascular insult [20,40]. ROS induces vascular angiogenesis by releasing VEGF. NOX enzymes result in the formation of superoxides. When these superoxides are combined with NO, a super reactive radical, peroxynitrite, is formed that can regulate VEGF and stimulate the new vessel formation [20].

Influence of micro-RNA in HIV infection and pulmonary vascular remodeling

Micro-RNA (mi-RNA) is a non-coding RNA molecule that consists of 21-24 nucleotides [41]. Widely used as a biomarker of various diseases, it is implicated in the pathogenesis of HIV-PAH [42]. Viruses regulate their machinery by integrating with the host genome for survival. The host mi-RNA can target the RNA viruses directly either by breaking them, stabilizing them, or inducing a change in the coded viral mi-RNA system. HIV can modulate the mi-RNA expression to promote its own replication and, in so doing, enhances the growth and survival in the host cell. The resulting HIV-specific mi-RNAs seem to activate an inflammatory response [43]. A pleiotropic mi-RNA, miR-21, which is essential for HIV-PAH pathogenesis, appears to be increased in HIV, HIV-PAH, and HCV co-infected patients compared to those not infected. A few animal models implicate miR-21 as one of the factors responsible for PAH. Others have demonstrated that miR-21 antagonists have a protective role against PAH [44]. Studies have shown that mi-RNAs, miR-27b, and miR-21 are increased in response to oxidative stress and regulate the macrophage actions by activating the NF-κB pathway, thereby increasing the pro-inflammatory mediators [42]. Also, there was upregulation of mi-RNA expression with reduced bone morphogenetic protein receptor-2 (BMPR-2) expression (a protein that inhibits smooth muscle growth and proliferation) from the ex-vivo findings in human lungs with HIV infections and illicit drug exposure [8].

**Figure 4:**
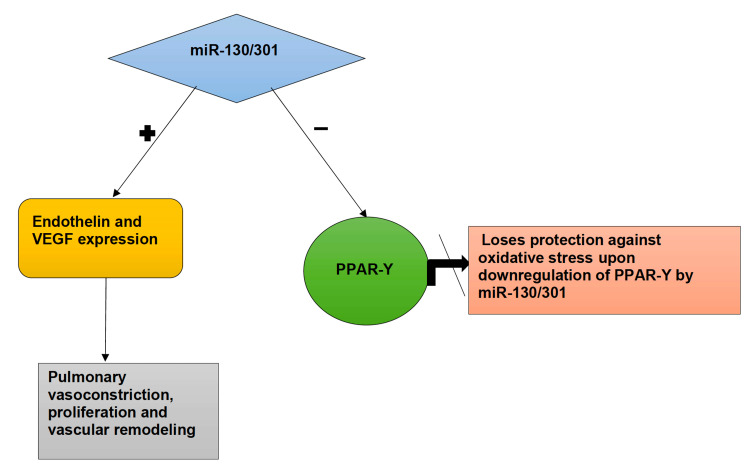
The role of mi-RNA on vascular remodeling PPAR-γ: peroxisome proliferator-activated receptor- γ; VEGF: vascular endothelial growth factor Image credit: Jaimee Jacob Palakeel.

Another family of mi-RNAs to be mentioned is the miR-130/301 family. During hypoxia-induced oxidative stress, the miR-130/301 family is upregulated. Each family member seems to potentiate the effect of another. The miR-130/301, when triggered, downregulates the expression of the peroxisome proliferator-activated receptor (PPAR-γ) [45]. PPAR-γ plays a vital role in vascular biology, from proliferation to extracellular matrix deposition. The upregulation of the PPAR-γ pathway functions as a protective factor against oxidative stress. PPAR-γ activates the Nrf2 pathway and binds to the antioxidant response element, protecting the lungs from oxidative stress. As mi-RNAs downregulate the activity of PPAR-γ, they lose their capability to protect the lung against oxidative stress [29]. It leads to increased endothelin-1 (EDN-1) and VEGF production with decreased expression of eNOS, increasing the pulmonary tone and cell proliferation. It subsequently results in Signal Transducer and Activator of Transcription 3 (STAT3) phosphorylation (a cytoplasmic latent transcription factor), leading to vascular remodeling, and ultimately PAH. The activated signaling pathways that miR-130/301 family induces cause extracellular matrix remodeling and cell proliferation, contributing to pulmonary vascular stiffness and, ultimately, PAH [45]. Figure 4 explains the influence of mi-RNA on vascular remodeling.

An outlook on inflammatory mediators like cytokines, growth factors, genetic predisposition, and associated risk factors predisposing PAH

PAH is characterized by changes such as endothelial proliferation, smooth muscle proliferation, medial hypertrophy, and fibrosis. Researchers have identified plexiform lesions in lung specimens of patients with PAH. Approximately 78% of patients with HIV-PAH demonstrate these changes. The lesions consist of perivascular inflammatory infiltrates more apparent in HIV-PAH [46]. Although idiopathic PAH occurs predominantly in females, HIV-PAH is seen more in males with a mean age of 33 years. Based on the findings of a study, the time interval from the diagnosis of HIV to the onset of PAH was around 33 months [47]. Since not all patients with HIV develop PAH, several studies have emphasized a genetic predisposition. Further studies on genetic predisposition may determine which individuals are susceptible to the disease. A study has shown the correlation of significant histocompatibility complex HLA-DR6 and HLA-DR52 with PAH. However, a few unpublished preliminary investigation reports have shown no association of these alleles with HIV-PAH patients [7]. Another gene that needs to be mentioned is the BMPR-2 [8,46,48,49]. The BMPR-2 negatively impacts smooth muscle growth and proliferation through intracellular signaling pathways of Suppressor of mothers against decapentaplegic (SMAD) proteins [8,46,49,50]. The heterozygous germline BMPR-2 mutation results in smooth muscle growth and proliferation, contributing to vascular remodeling. In the heterozygous BMPR-2+/- mice vs. wild-type mice study, the BMPR2+/- mice did not develop PAH. But when infused with serotonin or inoculated with an adenovirus, PAH developed, suggesting the influence of BMPR-2 mutation on the development of the disease [46]. Also, both HIV-Tat and cocaine downregulate BMPR- 2, leading to vascular SMC proliferation [8,50]. 

Recruitment of Inflammatory Cytokines and Adhesion Molecules in HIV-PAH

Activated inflammatory cells have been detected in immunohistochemistry of plexiform lesions, but not on regular stained sections [46]. The influx of inflammatory cells is seen around the perivascular spaces and plexiform lesions [50], namely, ECs, SMCs, macrophages, T-cells, and dendritic cells. Macrophage-released cytokines such as IL-6, IL-1β, TNF-α, and MIP-1 play a significant role in recruiting inflammatory cells [11,51-53]. IL-6 is a potent cytokine seen in the lungs of human PAH [11]. Steiner et al. reported overexpression of IL-6 in mice, similar to lesions seen in advanced PAH [27,54]. These plexiform lesions are induced by IL-6 and other upregulated factors like VEGR, transcription factors like cellular-myelocytomatosis oncogene (c-MYC), and MYC-associated factor X (MAX), and anti-apoptotic proteins survivin, and B-cell lymphoma-2 (Bcl-2) protein [27]. The HIV-Tat and cytokines IL-6, IL-1β, and TNF- α, act on endothelium, stimulating the p38 mitogen-activated protein kinases (MAPK) and NF-κB pathway, inducing ROS production. These result in gene and protein expression, increasing the release of vascular cell adhesion molecule (VCAM), intercellular adhesion molecule (ICAM), and more cytokines. The ongoing process leads to further monocyte recruitment, progressive inflammation, and repercussion of vascular injury [17,55].

Growth Factors Involved in HIV-PAH

Several growth factors, such as VEGF, PDGF, FGF, and epidermal growth factor (EGF), play a pivotal role in the migration, proliferation, and angiogenesis of ECs and SMCs [51]. Out of all, VEGF is the most critical factor that regulates angiogenesis. Many ligands, namely, HIF-1α, HIF-2α, estrogen, NF-κB, PPAR-γ, etc., influence the expression of VEGF. Upon binding to the VEGF receptor (VEGFR), upregulation of VEGF occurs, which results in kinase-dependent phosphorylation reactions such as the PI3K/AKT pathway and extracellular signal kinases activation, thus ultimately blocking apoptosis and promoting cell proliferation and survival [56]. Cool et al.illustrated the expression of VEGF in the ECs of the plexiform lesions, thus proving the prime role of VEGF in angiogenesis, increased permeability, and inflammation in the vascular endothelium [51,56]. Another growth factor is PDGF, enabling the migration and proliferation of fibroblasts and SMCs. The enhanced expression of PDGF-A mRNA was analyzed on the lung biopsy specimens collected from 13 HIV seronegative patients and one HIV seropositive patient using the semiquantitative polymerase chain reaction (PCR) technique. Similarly, immunohistochemistry has demonstrated an increased PDGF protein expression around perivascular spaces, suggesting its role in altering the pulmonary arterial wall [57]. Likewise, EGF release mediated by the extracellular matrix protein Tenascin-C favors cell proliferation, migration of SMCs, and disease progression [51].

The Action of Chemokines in Vascular Remodeling

A few essential chemokines mediate the inflammatory response and leukocyte recruitment. Fractalkine or chemokine C-X3-C motif ligand 1 (CX3CL1) is a chemoattractant synthesized by ECs, and its specific receptor, CX3CR1, is expressed in immune cells, like monocyte, macrophage, natural killer cells, and T-cells [46]. Perros et al. illustrated the presence of CX3CL1 surrounding the SMCs and plexiform lesions. In the rat model, pulmonary artery SMCs showed increased expression of CX3CL1 and its receptor, indicating its proliferative activity but not migratory function, emphasizing it as a growth factor for SMCs. Later, Sanchez et al. suggested that cell migration was mediated by another chemokine, C-C motif chemokine ligand 2 (CCL2), a monocyte chemotactic protein (MCP-1), which pulmonary ECs synthesized and were expressed abundantly in PAH [51].

Illegal drugs, Infections, and their significance in HIV-PAH

People who abuse drugs like cocaine, morphine, or other stimulants jeopardize developing HIV infection and its complications. Many animal studies, especially the SIV-infected morphine-treated macaques, when exposed to HIV proteins and illicit drugs, showed an upregulated trend in the release of IL-8 and MCP-1. These have demonstrated changes in the pulmonary vasculature with medial thickening of the pulmonary artery and plexiform lesions, infiltration of inflammatory cells within the intima, perivascular inflammation, and wholly obstructed vessels in PAH patients [58]. A study showed that exposure to morphine and HIV proteins led to initial endothelial apoptosis followed by the development of apoptotic resistant angio-proliferative HPMVECs. It implies a transition from initial VEGFR knockout followed by activation of VEGF by phosphorylation on chronic exposure to morphine and HIV proteins [59]. 

Certain known viral infections have shown their influence in accelerating HIV-PAH progression. HCV co-infection has been known to worsen the complications of HIV and is believed to contribute to PAH, either by viral toxicity in the lung vessels or through Portopulmonary hypertension. HCV works through dysregulation of host cell mi-RNAs, advancing the disease process during HIV/HCV co-infection [44]. Another virus, the human herpesvirus-8 (HHV-8), known to cause Kaposi sarcoma, is commonly involved in HIV infection [22,60]. It has been demonstrated that HHV-8 can infect cells, mainly the HPMVECs, and affect angiogenesis, inflammation, and apoptosis pathways. The infection causes alteration in gene expression and downregulates the BMPR-2 pathway leading to an apoptotic resistant phenotype considered necessary for the pathogenesis of PAH [60]. However, the association between HHV-8 and PAH has not been found yet. So, it is not clear whether HHV-8 is a causal factor [22].

Limitations

In this review, we collected all the articles from the PUBMED database, which were focused mainly on animal studies, with a few research on HPMVECs. We could not find data conducted on large-scale HIV patients that determined the association of risk factors such as drug use, HCV co-infection, gender, ethnicity, genetic diseases, duration of HIV illness, and the development of PAH. The importance of genetic predisposition in HIV-PAH was not clearly established. The study describes the effect of BMPR-2 mutation on smooth muscle proliferation and vascular remodeling but does not show the actual association between BMPR-2 mutation in HIV and the development of PAH. This study also describes the effect of HHV-8 on HIV-PAH but fails to deliver a true association. We addressed the role of HIV-Nef protein on EC proliferation and the formation of plexiform lesions. However, we could not delineate why it disrupts the Golgi functions and how that could contribute to PAH.

## Conclusions

This study aimed to identify potential risk factors involved in HIV-PAH pathogenesis and elucidate possible mechanisms. We were able to provide an outline of a few processes that were associated with HIV-PAH. Genetic factors may play a role in HIV-PAH, but the effect of BMPR2 mutations is still unknown. The association between BMPR-2 and PAH needs to be further investigated. Previous studies have shown that NOX enzymes produce ROS and modify arteries, so it will be necessary to validate and examine the effects of NOX inhibitors on HIV-PAH in more detail in the future. It is shown that HCV coinfection leads to vascular remodeling, so early detection and treatment are crucial for preventing PAH. Several studies have demonstrated the benefits of PPAR-γ and how mi-RNAs affect its activity. It is necessary to conduct a more comprehensive study on PPAR-γ as a remedy for HIV-PAH. To our knowledge, there is a lack of data representing the benefits of adding anti-inflammatory drugs and antioxidants to highly active antiretroviral therapy (HAART). Future research on this area must be considered as inflammation and oxidative stress are the crucial underlying factors responsible for the development of PAH. The influence of HIV proteins in anti-apoptotic angio-proliferative cellular state and vascular remodeling have been discussed in several studies, and this could inform future studies for targeted therapy in PAH.
